# Palladium Nanoparticles Synthesized by Laser Ablation in Liquids for Antimicrobial Applications

**DOI:** 10.3390/nano12152621

**Published:** 2022-07-29

**Authors:** Mónica Fernández-Arias, Ana M. Vilas, Mohamed Boutinguiza, Daniel Rodríguez, Felipe Arias-González, Pablo Pou-Álvarez, Antonio Riveiro, Javier Gil, Juan Pou

**Affiliations:** 1 LaserON Research Group, CINTECX, School of Engineering, University of Vigo, Lagoas-Marcosende, E-36310 Vigo, Spain; anamvilas@alumnos.uvigo.es (A.M.V.); mohamed@uvigo.es (M.B.); ppou@uvigo.es (P.P.-Á.); ariveiro@uvigo.es (A.R.); jpou@uvigo.es (J.P.); 2 Galicia Sur Health Research Institute (IIS Galicia Sur), SERGAS-UVIGO, E-36310 Vigo, Spain; 3 Biomaterials, Biomechanics and Tissue Engineering Group, Materials Science and Metallurgical Engineering Department, UPC-Barcelona TECH, 08930 Barcelona, Spain; daniel.rodriguez.rius@upc.edu; 4 School of Dentistry, Universitat Internacional de Catalunya, 08017 Barcelona, Spain; farias@uic.es (F.A.-G.); xavier.gil@uic.cat (J.G.)

**Keywords:** palladium nanoparticles, laser ablation, physicochemical characterization, bactericidal activity, cytocompatibility

## Abstract

Antibiotic resistance is a leading cause of death worldwide. In this paper, we explore new alternatives in the treatment of infections. Noble metal nanoparticles could help to mitigate this problem. In this work, palladium nanoparticles were synthesized by laser ablation in order to explore their antimicrobial capacity. To obtain palladium nanoparticles, a palladium plate immersed in water, or methanol, was ablated, using two pulsed lasers that emit radiation with wavelengths of 532 nm and 1064 nm, respectively. Pure Pd-NPs with crystalline microstructure and rounded shape were obtained. The nanoparticles’ size is more homogeneous if the laser wavelength is 532 nm, and it decreases when methanol is used as solvent, reaching mean diameters smaller than 6 nm. With the objective of studying antimicrobial activity against *Staphylococcus aureus*, the Pd-NPs were immobilized on the surface of titanium discs. The release of palladium ions was recorded during the first seven days, and the cytotoxicity of the immobilized NPs was also tested with L929 mouse fibroblast cell line. Palladium nanoparticles synthesized by means of the infrared laser in methanol showed a strong inhibitory effect on *S. aureus* and good cytocompatibility, with no toxic effect on fibroblast cells.

## 1. Introduction

One of the main problems that state health systems face today is the resistance of microorganisms causing infection (such as bacteria) when they are exposed to a drug that would normally kill them, or stop their growth (i.e., antibiotics), better known as antimicrobial resistance (AMR). The AMR is a result of the capacity of bacteria to change over time and adapt to specific drugs [1,2]. As a consequence, antibiotics designed to treat bacterial infections are less and less effective, and infectious diseases have become one of the leading causes of death in the 21st century [3,4]. On this point, in 2014 the World Health Organization (WHO) notified that 700,000 people were dying due to AMR, warning that this number could continue to grow, reaching 10 million in 2050 if additional bactericidal means were not developed [5]. More recently, a new study has reported a total of 929,000 deaths attributable to AMR during 2019, confirming the worst-case scenario [1].

Among all pathogens, *Staphylococcus aureus* is one of the biggest threats as being one of the most common bacteria responsible of skin infections and respiratory infections, such as pneumonia, but also for other serious infections such as endocarditis and osteomyelitis [6]. This gram-positive bacteria, whose resistance to penicillin was notified in 1942 [4], and later to methicillin during the 1960s [3,7], is nowadays one of the major multidrug-resistant pathogens to usual antibiotic treatment [3] and responsible for 100,000 deaths each year [1]. These reports highlight the necessity for finding an alternative to antibiotics as a way to treat diseases caused by bacteria [5]. 

The antibacterial properties of noble metals have been well known since ancient times, when gold and silver were commonly used to disinfect stored water, or to promote wound healing [8,9,10]. The use of noble metals, such as silver, in the nanometric range is of special interest. On the nanometric scale, the amount of surface per unit volume is enormous, so that properties of the nanoparticles of the same material are very different from bulk material [11]. Ag nanoparticles allow the properties of this metal to be enhanced, thus achieving a greater release of Ag ions and greater contact with the surrounding environment, in addition to facilitating their penetration through cell walls to attack microorganisms from the inside [12,13,14,15]. Unfortunately, resistance of different bacteria to silver nanoparticles has been reported recently [16], hence an alternative material to silver is required. 

In order to diversify treatments and avoid, as far as possible, the development of resistances, new approaches are focusing on noble metal nanoparticles. In this regard, palladium is one of the six platinum-group elements, a noble metal whose extraordinary catalytic activity [17,18,19,20] and capacity to stabilize a wide diversity of matter, confers it great versatility, being commonly used in the biomedical field as part of cancer therapy, in dentistry devices, and in probes for analyte detection, among others [21,22]. In recent years, its outstanding antibacterial capacity has also attracted widespread interest [22,23,24]. For these antimicrobial applications, characteristics such as size, morphology, crystallinity, or oxidation state of the nanoparticles are of great importance [14].

Among the different methods for producing nanoparticles, laser ablation of solids in liquids (LASL) makes it possible to obtain NPs without any precursor or chemical reaction, which can produce harmful byproducts [25,26,27]. This point is crucial, especially when the objective of these nanoparticles is to use them in biomedical applications [28,29]. Additionally, the laser ablation technique has been shown to allow both size and shape control, by adjusting processing parameters [30,31]. Although different palladium nanoparticles have been previously obtained by laser ablation in liquid [32,33,34,35,36,37,38,39,40,41,42,43,44,45,46], to the knowledge of the authors, their bactericidal capacity has not been studied before.

This work reports on the synthesis of palladium nanoparticles by the LASL technique, and their antimicrobial activity. Palladium nanoparticles were obtained by laser ablation of a Pd target in water and methanol, using two different laser sources working at two different wavelengths. Antibacterial activity of Pd nanoparticles was studied with *Staphylococcus aureus,* and the cytotoxicity was evaluated with a fibroblast cell line to study the ability of the nanoparticles to kill bacteria, without damaging the surrounding healthy tissues.

## 2. Materials and Methods

### 2.1. Laser Ablation

A palladium foil with 99.99% of purity (Thermo Fisher Scientific, Waltham, MA, USA) was gently cleaned with methanol and rinsed with ultrapure deionized water. After this careful cleaning, the Pd foil was used as target for the laser ablation experiments. Tests were carried out with two laser sources emitting radiation at two different wavelengths (532 and 1064 nm) in two liquid media, resulting in 4 samples as indicated in Table 1.

**Figure 1:**
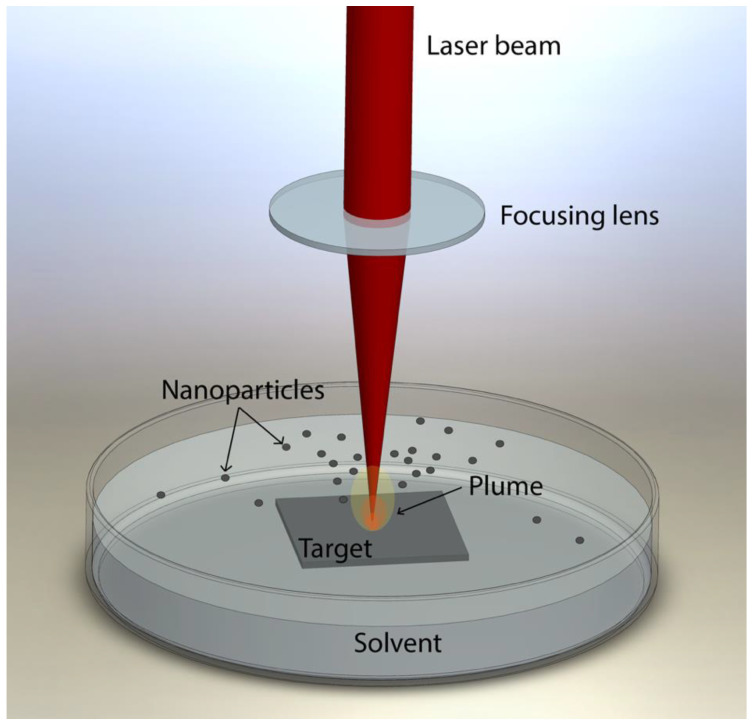
Laser ablation process.

The target was submerged in 100 mL of liquid (ultrapure deionized water or methanol), keeping its upper surface covered by 1 mm of liquid. The ablation process is sketched in Figure 1.

The main laser processing conditions are reported in Table 2. 

The laser sources used were two diode-pumped Nd:YVO4 lasers (Rofin-Sinar, Hamburg, Germany) emitting radiation at 532 nm and at 1064 nm of wavelength, respectively, with a frequency of 20 kHz. The green laser provided 14 ns long pulses of 0.26 mJ of energy, being of 20 ns and 0.36 mJ in the case of the infrared laser. During the ablation process, the laser beam was kept focused on the palladium target surface, moving the laser spot across the surface at a speed of 50 mm/s. The processing time for the LASL of each sample was the one required for obtaining 2 mg of palladium nanoparticles.

### 2.2. Colloidal Nanoparticles

#### Physicochemical Characterization

After each laser ablation process, drops of the synthesized colloidal solutions were carefully placed on Formvar/Carbon supported copper grids with 400 mesh of size (Ted Pella Inc., Redding, CA, USA) and left to dry before proceeding to their analysis. Particle size, morphology and dispersion were characterized by transmission electron microscopy (TEM) using JEOL JEM 1010 equipment (JEOL, Akishima, Japan), and field emission scanning electron microscopy (FESEM) using a JEOL JSM 6700F microscope (JEOL, Akishima, Japan). Size distribution of each type of sample, as seen in to Table 2, was studied using the TEM micrographs obtained. For this, the diameter of 400 particles of each type was measured. The palladium nanoparticles were also observed by high resolution transmission electron microscopy (HRTEM) using JEOL JEM 2010F FEG (JEOL, Akishima, Japan), in order to study their crystalline structure.

Drops of each type of sample were also placed on glass sample holders to obtain a film with the required thickness to be analyzed by X-ray diffraction (XRD). These XRD analyses allowed us to verify the crystal structure obtained by HRTEM. The Pananalytical X’Pert Pro X-ray diffractometer (Malvern Panalytical, Malvern, UK), using monochromated Cu-Kα radiation (λ = 1.54 Å) over the 30–100° 2θ range with step size of 0.026°, was used to perform the XRD analysis. The XRD spectra of each sample was compared to that of the precursor material (palladium foil) and the ICDD-JCPDS database used to identify the crystalline phases.

The UV–VIS absorption spectrum, measured from 190 to 790 nm with a HP 8452 spectrophotometer (Hewlett Packard, Palo Alto, CA, USA), was used to study the optical properties and stability of the colloidal suspensions. 

### 2.3. Immobilized Nanoparticles

With the objective of verifying that the synthesized nanoparticles are effective against bacteria without damaging healthy tissues, bacterial adhesion assay and cytotoxicity study was performed. The ion release kinetics was also conducted to elucidate the effect of palladium ions in the antimicrobial process. 

To perform these assays, the obtained Pd-NPs were deposited on commercially pure titanium (cp Ti) discs. Among all titanium and its alloys, cp Ti grade 2 is one of the most widely used materials in biomedical applications, such as dental implants [47,48]. Therefore, discs of grade 2 titanium (Goodfellow Cambridge Limited, Huntingdon, UK) with an average surface roughness of about 200 nm, were used as substrates. These titanium discs were previously cleaned in an ultrasonic bath with methanol for 15 min and rinsed with ultrapure deionized water to remove any possible contamination. After that, discs were submerged in 25 mL of each condition (see Table 1), allowing the liquid to evaporate at room temperature. As result of evaporation, the nanoparticles remain immobilized on the surface of the disc, forming a coating.

Additionally, 3 discs were set apart to be used as control for each type of test. 

#### 2.3.1. Ion Release

The kinetics of palladium ion release from the Pd-NPs immobilized on the surface of titanium discs was tracked for 7 days. Three replicates (treated discs) of each condition, and three untreated discs as control, were taken separately, each one in a different sterile specimen container and immersed in 25 mL of deionized ultrapure water to measure the ion release.

Afterwards, 1.5 mL of each container were collected periodically during 7 days in Eppendorf tubes. The tubes were subjected to centrifugation using an Eppendorf miniSpin equipment (Eppendorf, Hamburg, Germany) at 13,400 rpm for 20 min at room temperature. This made it possible to ensure the absence of nanoparticles when measuring the release of ions. The ion content was measured using Optima 4300 DV (Perkin Elmer, Waltham, MA, USA) equipment for inductively coupled plasma optical emission spectrometry (ICP-OES). To do this, after centrifugation, 1 mL of the upper area of each Eppendorf tube was analyzed. The extracted volume was filled with fresh deionized water after sampling, to maintain an unchanged volume.

#### 2.3.2. Bacterial Growth Inhibition

The effect of the presence of nanoparticles on the adhesion of bacteria to the titanium surface was studied with *S. aureus* (Spanish Collection of Type Cultures (CECT) 435, Spain) cultured in BHI broth (Scharlab SL, Sentmenat, Spain). A bacterial inoculum was incubated at a temperature of 37 °C for 24 h prior to testing. The optical density of the bacteria suspension was adjusted to 0.2 ± 0.01 at 600 nm, which is equivalent to 1 × 10^8^ colony-forming units (CFU)/mL. Both the control samples (without nanoparticles) and those containing the nanoparticles were immersed in two baths of ethanol and distilled water for 15 min each and placed in a 24-well plate (Nunc, Rochester, NY, USA) with 1 mL of bacterial suspension at 37 °C for 2 h. In order to remove non-adherent bacteria, the samples were subsequently washed three times with phosphate buffered solution (PBS). The samples were sonicated in 1 mL of sterile PBS for 5 min, in order to collect the adherent bacteria. The phosphate buffered solution was sequentially diluted. Next, the diluted bacterial suspensions were seeded on agar plates supplemented with nutrient rich BHI medium. The following step was the incubation of the agar plates at a temperature of 37 °C for 24 h and, subsequently, the colony forming units (CFUs) were counted. Samples were fixed with 2.5% glutaraldehyde in PBS for 1 h at 4 °C. Afterwards, samples were dehydrated by immersing them successively in ethanol series (50%, 70%, 96% and 100%). The presence and morphology of bacteria on the surfaces was examined with a Phenom XL Desktop scanning electron microscope (PhenomWorld, Eindhoven, The Netherlands), at a potential of 20 kV. Three replicates of each type of sample were used in this assay.

#### 2.3.3. Cytotoxicity

The immobilized Pd-NPs with the highest bacterial inhibition and statistical significance were selected to perform the cell viability and proliferation tests, by means of XTT cell proliferation assay (Canvax, Córdoba, Spain). Two titanium discs of each condition and controls (untreated titanium discs), were sterilized with ethanol and UV, to be placed in a P24 culture plate and immersed in 400 µL of laminin at 10 µg/mL at 4 °C overnight. Subsequently, the discs were washed with PBS in order to remove the laminin. At the same time, the L929 (NCTC clone 929 of strain L) cell lines were thawed in a T-75 flask for proliferation. After being thawed, the L929 cells were seeded on the titanium discs with 1 mL of culture medium (DMEM (Gibco) with supplement of GlutaMAX, fetal bobine serum (10%), penicillin/streptomycin (1%) and 0.1% amphotericin B (0.1%)) at an approximate concentration of 180,000 cells/mL.

The culture medium was removed from each seeded well after 24 h and 50 µL of trypsin added to detach the cells. After 3 min at 37 °C, the trypsin reaction was stopped by adding 300 µL of fresh culture medium. The entire volume of each well was collected and transferred to a P96 reading plate, acquiring 3 replicate wells of each plate (6 replica of each sample and 3 replica of the control) of 100 µL each one. Subsequently, wells are left in incubation for 2 h, so that the living cells adhere to the plate. After that, 50 µL of XTT reagent mixture (0.1% of reagent plus 2% of activator) were added to each well. The wells are left for 2 h in the incubator at 37 °C in CO_2_ atmosphere. After shaking gently for 1 min on a rocking shaker, the absorbance (A) is measured in a FLUOstar Omega microplate reader (BMG Labtech, Ortenberg, Germany), to evaluate the cell proliferation. The wavelength used was 450 nm, against a reference wavelength of 650 nm, to detect the stained (alive) cells [49,50]. The absorbance values obtained at 24 h for each of the 6 replicates were used to calculate mean absorbance of each condition. 

#### 2.3.4. Statistical Analysis

The information acquired through the bacterial adhesion study and the cytotoxicity test was statistically analyzed following the procedures previously reported by the authors [51]. The quantitative data were expressed as mean ± standard deviation (SD), and the difference between the mean of each group and the mean of the control group was accepted as statistically significant when *p*-value < 0.05.

## 3. Results and Discussion

Pd-NPs in colloidal solution were successfully produced by means of the two lasers tested, one delivering radiation at 532 nm and the other at 1064 nm, and in both liquids: water and methanol.

### 3.1. Characterization of the Nanoparticles in Solution

**Figure 2:**
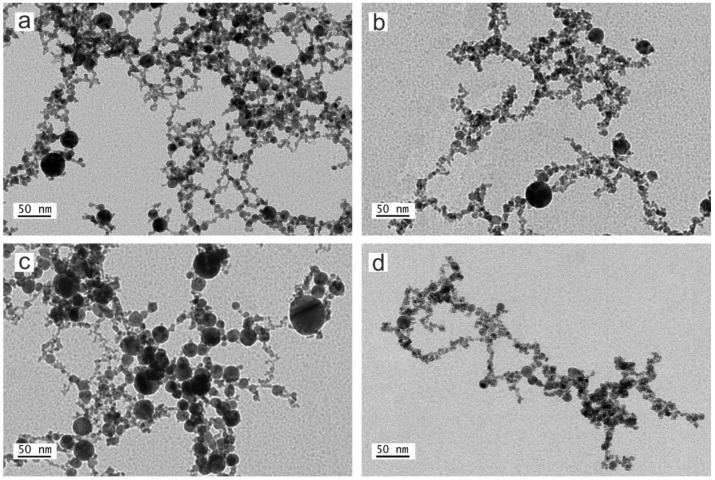
TEM micrographs of Pd nanoparticles synthesized by laser ablation with (**a**) 532 nm of wavelength in water, (**b**) 532 nm in methanol, (**c**) 1064 nm in water and (**d**) 1064 nm in methanol.

**Figure 3:**
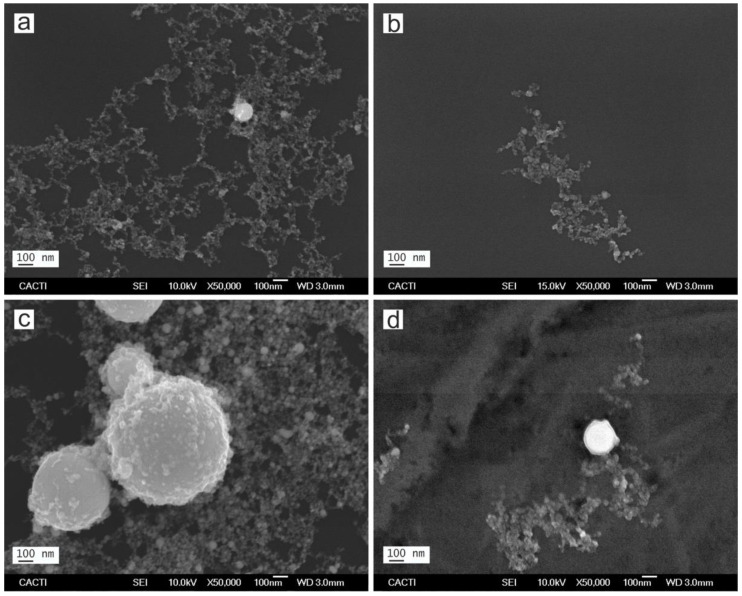
FESEM micrographs of Pd nanoparticles synthesized by laser ablation with (**a**) 532 nm of wavelength in water, (**b**) 532 nm in methanol, (**c**) 1064 nm in water and (**d**) 1064 nm in methanol.

The morphology and size distribution evaluation of the nanoparticles synthesized by laser ablation was carried out by means of different TEM and FESEM electron microscopy images. Figure 2; Figure 3 show representative micrographs of the appearance of the nanoparticles, obtained by TEM (Figure 2) and FESEM (Figure 3).

Note that all the Pd-NPs obtained by LASL are spherical, showing a certain trend to agglomeration forming string-like structures. This is due to the thermal regime between laser beam and target during the formation process. This regime depends on the laser parameters and the target features, but the specific properties of the solvent are decisive in the process [31,52]. 

The energy carried by the laser beam is transmitted to the uppermost layers of the material when the laser beam hits its surface. This produces an increase in temperature, raising it above its melting point. Since the high power is delivered in a few nanoseconds of pulse, the energy density exceeds the ablation threshold of the material, leading to surface melting, vaporization, and material ejection. In this process, different species such as ions, molecules and particles are emitted from the surface, absorbing the incoming energy, and resulting in plasma formation [30,39,53]. When the process takes place in liquid, the plasma plume is constrained and its temperature decreases swiftly, forming spherical nanoparticles [30]. 

**Figure 4:**
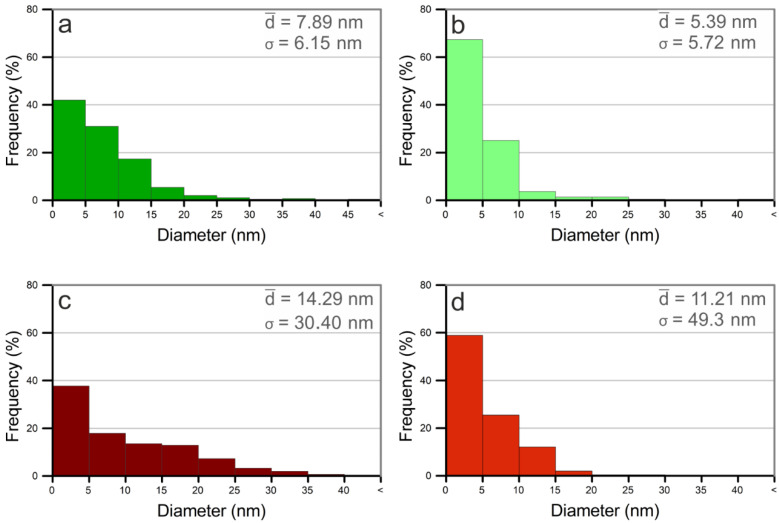
Size distribution of Pd nanoparticles synthesized by laser ablation with (**a**) 532 nm of wavelength in water, (**b**) 532 nm in methanol, (**c**) 1064 nm in water and (**d**) 1064 nm in methanol.

As can be seen from Figure 4, the nanoparticles synthesized in methanol have smaller dimensions than those synthesized in water, revealing the important role of the solvent in nanoparticle formation and growth. As previous works have revealed, the presence of carbon from organic solvents such as methanol, has a crucial role in the nucleation and growth of nanoparticles [54]. While in water, oxidation of the ejected material is promoted by molecular oxygen, the carbon species (*C) from methanol, acts to protect the surface of the incipient particles, stopping the growth process and preventing agglomeration. As a result, the dimensions of the obtained Pd-NPs produced in the organic solvent present a smaller average size than those synthesized in water, using the same processing conditions [37,52]. This reduction in the average size of the nanoparticles obtained in organic solvent is in agreement with the results obtained with Pd by other authors [33], and by our group with other metal, such as Cu [51].

Regarding the laser parameters, it is known that different mechanisms such as penetration depth of the laser beam, self-absorption, ablation efficiency, etc. are related to the laser wavelength, and have a great influence in the results. In particular, the absorption efficiency of the incident laser energy determines, to a large extent, the nanoparticles’ size and/or concentration of the obtained nanoparticles. In this regard, it is known that the absorption efficiency in the laser ablation of Pd-NPs is higher at long wavelengths [34]. Thus, in the nanosecond pulse duration, the plasma heating is much higher at 1064 nm than at 532 nm. This contributes to the formation of some larger nanoparticles [34,39] and, as a result, Pd-NPs obtained with 1064 nm present a broader size dispersion than those obtained with 532 nm.

The TEM and FSEM images of the obtained nanoparticles corroborate this fact, and it can be clearly observed by means of the corresponding histograms (see Figure 4). The diameter of approximately 400 nanoparticles was measured utilizing different TEM electron microscopy images. These diameter values are shown as histograms in Figure 4. 

Additionally, it is noteworthy to mention that in accordance with previous works, the effect of the carbon species (*C) makes the ablation process comparatively much slower, requiring more time to reach the same concentration of NPs than when water is used as solvent [35].

**Figure 5:**
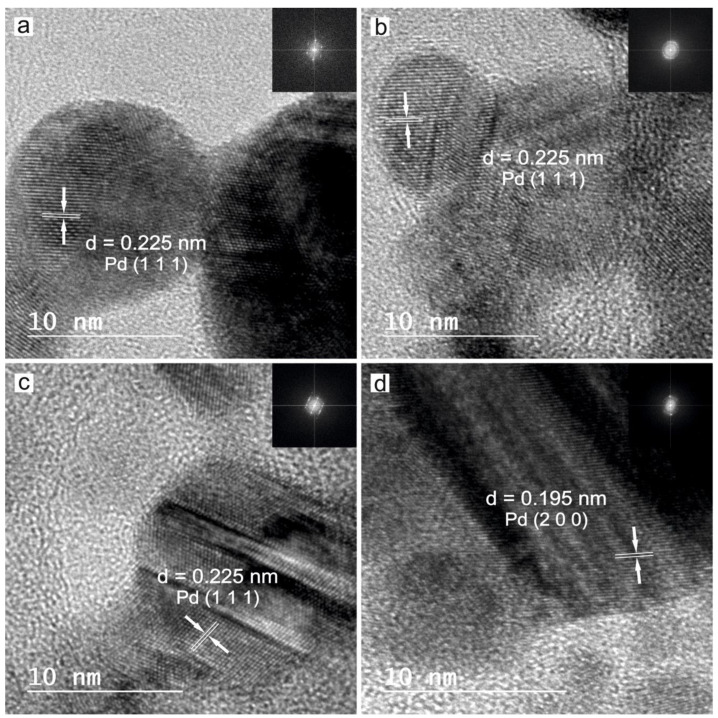
HRTEM of Pd nanoparticles synthesized by laser ablation with (**a**) 532 nm of wavelength in water, (**b**) 532 nm in methanol, (**c**) 1064 nm in water and (**d**) 1064 nm in methanol.

With the objective of identifying the crystalline phases of the laser synthesized Pd nanoparticles, HRTEM analysis was performed on each sample, revealing that all the obtained nanoparticles are crystalline. This feature is clearly evident in Figure 5, where the HRTEM images show the lattice structure of the palladium nanoparticles, and their corresponding Fast Fourier Transform (FFT).

The measured interplanar distances, and the corresponding FFT, resulted in 0.225 nm and 0.195 nm, which corresponds with the (111) and (200) family planes of metallic Palladium (JCPDS-ICDD ref.00-005-0681).

**Figure 6:**
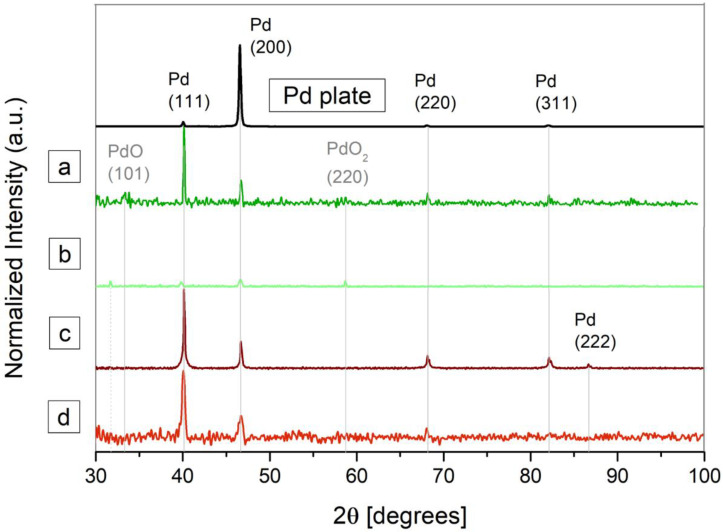
XRD of the palladium target and palladium nanoparticles synthesized by laser ablation with (**a**) 532 nm of wavelength in water, (**b**) 532 nm in methanol, (**c**) 1064 nm in water and (**d**) 1064 nm in methanol, comparing the diffraction pattern of the palladium plate used as target. The position of the representative diffraction peaks is indicated by the gray lines.

With the aim of providing a more exhaustive analysis on the crystalline phases, XRD was carried out on the nanoparticles, but also on the palladium foil (target) for a comparative analysis. Figure 6 shows the diffraction patterns of the four groups of samples under study.

Figure 6 evidences that the diffraction pattern of the palladium plate is consistent with typical face-centered cubic (FCC) of metallic palladium (JCPDS-ICDD ref.00-005-0681), showing higher relative peak intensity at 46.66°, which could be indicative of crystallographic preferred orientation in the (200) direction. 

The XRD analysis confirmed the elemental nature of the Pd-NPs synthesized. All are crystalline and pure palladium with characteristic diffraction peaks (111), (200), (220) and (311) at 2*θ* values of 40.12°, 46.66°, 68.09° and 86.6°, respectively. Only those obtained in water with 532 nm of wavelength (sample a) exhibit a very low degree of oxidation. In this case, peaks at 2*θ* values of 33.89° and 58.2° corresponding with PdO (JCPDS-ICDD ref.00-043-1024) and PdO2 (JCPDS-ICDD ref.00-034-1101), respectively, can be observed. In a deeper analysis, the presence of carbon can be observed when methanol is used as solvent. On one hand, the broad shoulder located at low angles in the case of sample d, is attributable to amorphous carbon [42]. On the other hand, the XRD peak at 32.29° present in the case of sample b, could be attributable to cubic C, according to the corresponding pattern (JCPDS-ICDD ref.00-018-0311). 

As expected, palladium nanoparticles synthesized with 1064 nm of wavelength in methanol (sample d), show wider diffraction peaks than the other three Pd-NPs groups. This effect, is related to the crystallite size or stress/strain in the crystal lattice. The XRD data allow determining the particle size *D*, by means of the Scherrer equation:

(1) $$D=\frac{K\lambda }{\beta cos\theta }$$

where *β* is the full-width at half maxima (FWHM) of the diffraction peaks. *θ* is the Bragg angle, K is the Scherrer constant and λ is the wavelength of the X-ray source. The width of the diffraction peak varies inversely with the particle size [22,42,55]. Calculations shown a size of 10.26 nm, which is consistent with de TEM analysis and the corresponding histogram obtained. 

Additionally, a stressed Pd structure caused by interstitial Pd alloys, is in line with typical results of laser ablation in methanol. When the ablation process takes place in organic solvents, C and H are available to form interstitial alloys. As pointed out by Giorgetti et al., adjacent peaks to those corresponding to (111) and (200) Pd, at 40.12° and 46.66°, respectively, would match with PdCx and PdHx [42]. 

It is noteworthy to mention that peak positions in the XRD pattern of the palladium plate match with those of the palladium nanoparticles obtained, but intensities are quite different. This type of behavior is commonly found in thin films, and revealed a certain preferential orientation of each sample [56].

The optical properties of the obtained Pd-NPs in solution were determined from their corresponding absorbance spectra, measured immediately after the generation process.

**Figure 7:**
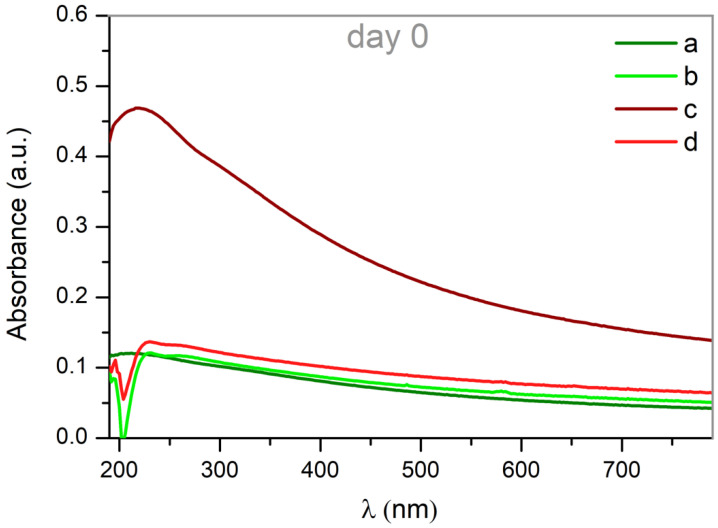
UV–VIS spectra of the as ablated palladium nanoparticles synthesized by laser with (**a**) 532 nm of wavelength in water, (**b**) 532 nm in methanol, (**c**) 1064 nm in water and (**d**) 1064 nm in methanol. The contribution due to the pure solvent has been subtracted from the spectra.

As shown in Figure 7, the UV–VIS spectra of the nanoparticles obtained in methanol (samples (b) and (d)), together with those synthesized in water with the infrared laser (sample (c)), exhibit a significant absorption band around 225 nm, which is related to the interband transition in metallic colloidal solutions. These typical spectra, can be caused by quantum size effects due to very small Pd-NPs [35,42]. Close inspection of these spectra reveals a shoulder around 258 nm, which could be due to the presence of carbon when methanol is used as solvent. These results corroborate those obtained by the XRD analysis.

In the case of the Pd-NPs synthesized by laser ablation in water with the 532 nm wavelength laser (sample (a)), the absorption band around 200 nm suggest the presence of ionic Pd^2+^ [33,37,39].

The stability of the Pd-NPs in suspension was studied by repeating the absorbance measurements of all samples after 180 days.

**Figure 8:**
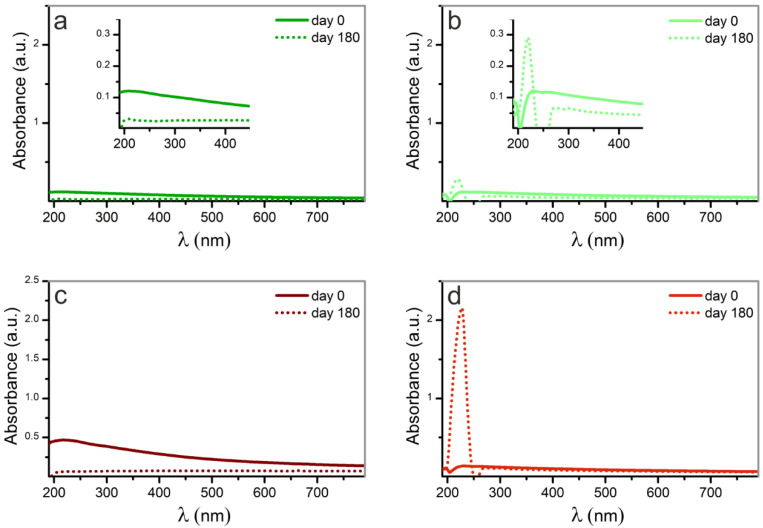
UV–VIS spectra of the Pd_NPs after 180 days, compared to the corresponding ones as ablated. Palladium nanoparticles synthesized by laser ablation with (**a**) 532 nm of wavelength in water, (**b**) 532 nm in methanol, (**c**) 1064 nm in water and (**d**) 1064 nm in methanol. Insets in figures (**a**,**b**) are enlargements of the zone of interest of the spectra.

As shown in Figure 8, there is a substantially different evolution of the absorbance among samples obtained in methanol, and those obtained in water. On one hand, the absorption band measured on day 0 in Pd-NPs synthesized in water (samples (a) and (c)), have been flattened and broadened slightly after 180 days, as sign of agglomeration. On the other hand, in the case of Pd-NPs obtained in methanol (samples (b) and (d)), the absorbance peak is surprisingly increased, which can be attributed to the high ability of Pd to lose electrons forming Pd^2+^. Additionally, a slightly blue-shift is also evident when we observe the absorbance of sample (b), which could be attributed to the formation of Pd Carbide. 

These spectral features are related to a molecular-like absorption behavior, or to solvent degradation [42]. The latter is related to the presence of amorphous carbon at the surface of the NPs, which is in good agreement with the results obtained in the XRD analysis. 

### 3.2. Ions Release Rate from the Nanoparticles Coating

The release of palladium ions from immobilized nanoparticles was measured throughout seven days. 

**Figure 9:**
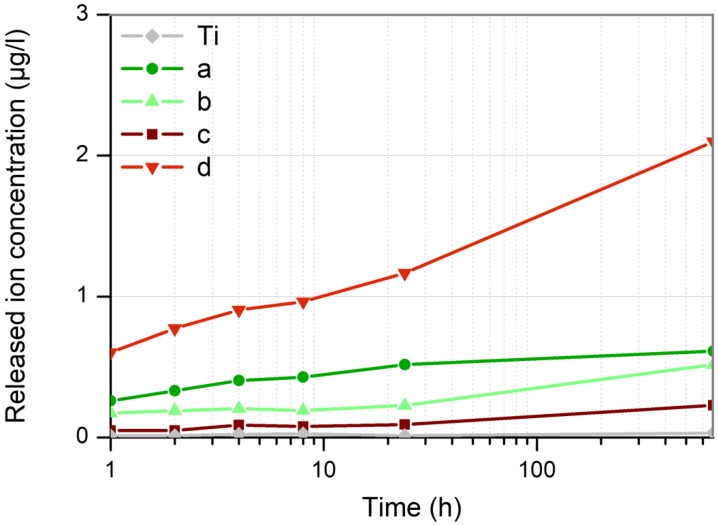
Palladium ions release kinetics from cp Titanium discs with immobilized Pd nanoparticles on their surface, obtained by laser ablation with (**a**) 532 nm of wavelength in water, (**b**) 532 nm in methanol, (**c**) 1064 nm in water and (**d**) 1064 nm in methanol.

As evident from the Figure 9, the palladium ion release rate of the Pd-NPs synthesized in methanol (samples (b) and (d)) increases over time, especially after 24 h. This release process is substantially higher when the NPs are obtained with 1064 nm of wavelength (sample (d)). In case of Pd-NPs obtained in water with 532 nm (sample (a)), the release rate increases slightly up to 24 h, and then remains constant over time. On its part, Pd-NPs synthesized in water with the infrared laser (sample (c)), shows minimal palladium ion release kinetics, very similar behavior to the control (titanium without NPs). 

### 3.3. Antimicrobial Activity

**Figure 10:**
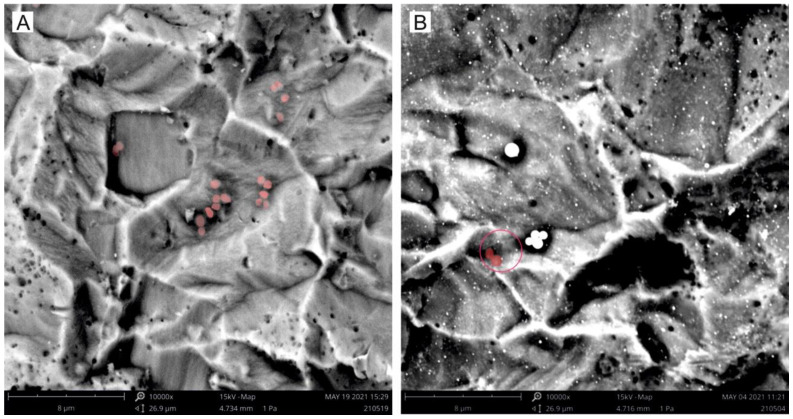
SEM images of bacterial adhesion on (**A**) bare titanium and (**B**) titanium with palladium NPs synthesized by laser ablation in water with 532 nm of wavelength. Bacteria are highlighted in false color red for clarity.

With the objective of exploring the toxicological properties of palladium NPs, the bacterial adhesion assay was carried out against *S. aureus*. The bactericidal activity of laser synthesized palladium nanoparticles can be qualitatively determined by means of SEM images taken after the bacteria adhesion test (see Figure 10). 

As seen in Figure 10, the presence of Pd-NPs immobilized on the surface of the titanium discs prevent most *S. aureus* from adhering to the surface. While the discs without palladium nanoparticles are a good environment for the bacteria to attach (see red dots in Figure 10A), just a few bacteria were found on the surface of the treated discs (note the red dots inside the red circle in Figure 10B). 

**Figure 11:**
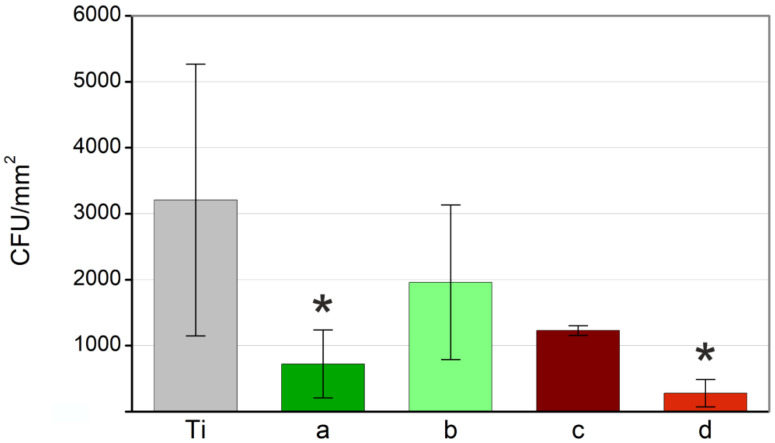
Bactericidal effect of the Pd nanoparticles synthesized with (**a**) 532 nm of wavelength in water, (**b**) 532 nm in methanol, (**c**) 1064 nm in water and (**d**) 1064 nm in methanol against S. aureus. Values presented as mean ± SD and significant difference of each group with the control group (* *p*-value < 0.05) marked.

Figure 11 shows the relative absorbance for all groups under study, and the control titanium sample without Pd-NPs. As described in Section 2.3.2, this absorbance is directly related to the colony-forming units, which allows evaluating quantitatively the bactericidal effect of palladium nanoparticles.

As can be observed in Figure 11, all the samples treated with Pd-NPs show an average antibacterial effect against *S. aureus*, compared to the control (titanium without NPs). However, no significant difference was found among the four experimental groups. Only Pd-NPs synthesized in (a) water with 532 nm of wavelength and in (b) methanol with 1064 nm, present a significant difference with the bare titanium. Despite the enormous influence that nanoparticles size has been shown to have in diverse applications, our results seem to indicate that the antimicrobial effect of Pd-NPs is not directly related to their size. Other parameters, such as stability and ion release kinetics, seem to have a higher influence on their toxicity.

To this day, there is still no general agreement about the mechanism used by NPs to kill bacteria. Several works prior to this one demonstrated that this mechanism is different, depending on the type of pathogen. Very different results were obtained under the same conditions (NPs with identical size, morphology, and composition) but different bacteria [22,23]. In our case, samples that exhibited the strongest bactericidal effect (d) and (a) against *S. aureus*, are those that experienced the highest Pd^2+^ ion release kinetics, as shown in Figure 9. 

### 3.4. Cell Viability Assay

With the objective of verifying that the obtained nanoparticles have effective bactericidal activity, but do not cause damage to healthy tissues, cell viability assays were carried out with L929 cell line fibroblast from mouse. The method used for this analysis was the live-dead staining by XTT. This colorimetric assay is based on the reduction of a yellow tetrazolium salt to an orange formazan dye, by cellular metabolic activity. As this conversion only occurs in metabolically active cells, the viable cells in the samples are proportional to the amount of formazan produced. Consequently, the viability of a sample can be quantified, measuring the absorbance at a wavelength of 450 nm against the absorbance of the background at 650 nm [49,50].

**Figure 12:**
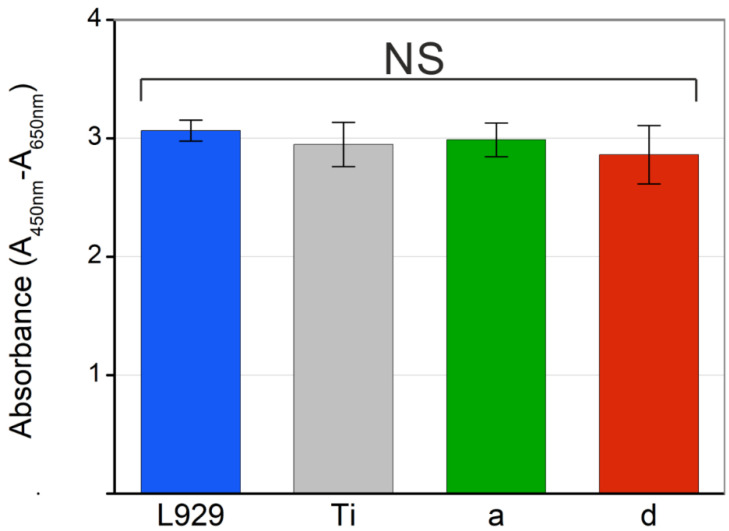
Viability of L929 fibroblast cells expressed in terms of normalized absorbance. (**L929**) control culture of fibroblasts; (**Ti**) culture of fibroblasts on titanium discs without nanoparticles; (**a**,**d**) culture of fibroblasts on titanium discs with immobilized Pd nanoparticles synthesized by laser ablation (**a**) in water with 532 nm of wavelength and (**d**) in methanol with 1064 nm.

Figure 12 shows results of fibroblast cells viability expressed in terms of absorbance. These results indicate that samples of titanium coated with Pd-NPs present statistically similar results to bare titanium.

Pd^2+^ ions are well known as being responsible for many enzymatic processes in eukaryotic, as well as prokaryotic cells [22]. In our case, analyzing the results obtained in the cell viability assay, the release of Pd^2+^ ions, does not seem to affect the cells. These results demonstrate that the use of nanometric-scale materials allows us to take advantage of their bactericidal properties, while preserving the integrity of healthy tissue that surrounds it [57].

These are promising results in the search for an alternative to Ag nanoparticles as bactericidal treatment, which allows preventing antimicrobial resistance. But much more research is necessary, including microbiologic tests with other bacteria (especially gram-negative), larger incubation times and biologic testing in animal models, as well as clinical trials, before these palladium nanoparticles could be safely used for human therapeutics.

## 4. Conclusions

Spherical palladium nanoparticles with crystalline structure have been successfully synthesized by the laser ablation of solids in liquids (LASL) technique, utilizing two lasers emitting at different wavelengths in two different solvents. The laser synthesized nanoparticles showed small size, reaching mean diameters smaller than 6 nm.

The bacterial adhesion assays performed, evidence the antimicrobial effectiveness of the Pd-NPs coating. *Staphylococcus aureus* was shown to be more susceptible to the nanoparticles synthesized with the IR laser in methanol. Pd^2+^ ions release seems to play a crucial role in the bactericidal activity of the nanoparticles obtained. 

On the other hand, the results obtained by means of the viability assay seem to reveal that the concentration of ions released by the palladium nanoparticles are enough to kill bacteria efficiently, but are below the limits necessary to produce harmful effects in cells.

The remarkable antimicrobial activity of the palladium nanoparticles against *Staphylococcuss aureus*, as well as their cytocompatibility, evidences their utility as a bactericidal factor, but more biologic tests both in vitro and in vivo, as well as clinical trials, are required prior to application in humans.

## Figures and Tables

**Table 1:** Types of samples studied in this work.

Sample	Laser Source	Liquid Medium
a	532 nm	Water
b	532 nm	Methanol
c	1064 nm	Water
d	1064 nm	Methanol

**Table 2:** Laser processing conditions with each laser source.

Wavelength (nm)	Pulse Duration	Pulse Frequency (kHz)	Pulse Energy (mJ)
532	14	20	0.26
1064	20		0.36

## Data Availability

Not applicable.
